# Dataset of inertial measurements of smartphones and smartwatches for human activity recognition

**DOI:** 10.1016/j.dib.2023.109809

**Published:** 2023-11-17

**Authors:** Miguel Matey-Sanz, Sven Casteleyn, Carlos Granell

**Affiliations:** GEOTEC Research Group, Institute of New Imaging Technologies, Universitat Jaume I, Castellón de la Plana 12071, Spain

**Keywords:** HAR, Mobile devices, Inertial sensors, Heterogeneous subjects, Cross-subject evaluation

## Abstract

This article describes a dataset for human activity recognition with inertial measurements, i.e., accelerometer and gyroscope, from a smartphone and a smartwatch placed in the left pocket and on the left wrist, respectively. Twenty-three heterogeneous subjects (*μ* = 44.3, *σ* = 14.3, 56% male) participated in the data collection, which consisted of performing five activities (*seated, standing up, walking, turning,* and *sitting down*) arranged in a specific sequence (corresponding with the TUG test). Subjects performed the sequence of activities multiple times while the devices collected inertial data at 100 Hz and were video-recorded by a researcher for data labelling purposes. The goal of this dataset is to provide smartphone- and smartwatch-based inertial data for human activity recognition collected from a heterogeneous (i.e., age-diverse, gender-balanced) set of subjects. Along with the dataset, the repository includes demographic information (age, gender), information about each sequence of activities (smartphone's orientation in the pocket, direction of turns), and a Python package with utility functions (data loading, visualization, etc). The dataset can be reused for different purposes in the field of human activity recognition, from cross-subject evaluation to comparison of recognition performance using data from smartphones and smartwatches.

Specifications Table

**Table:** 

Subject	Applied Machine Learning.
Specific subject area	Human activity recognition based on inertial sensors.
Data format	Raw
Type of data	Table (.csv)
Data collection	Accelerometer and gyroscope samples were collected at 100 Hz using a smartphone (Xiaomi Poco X3 Pro) and a smartwatch (TicWatch Pro 3 GPS). The devices were given to the 23 participants, who were instructed to carry the smartphone in their left pocket and the smartwatch on their left wrist. Both devices had custom data collection applications installed that stored the samples on the device. Each participant performed a sequence of activities consisting of standing up from a chair, walking three meters, turning around, walking back to the chair and sitting down on the chair. The data collection process was video-recorded at 60 frames per second using a Xiaomi Poco F2 Pro to manually label the collected data.
Data source location	• Institution: GEOTEC Research Group, Universitat Jaume I • City/Town/Región: Castellón de la Plana, 12071 • Country: Spain
Data accessibility	Repository name: Zenodo Data identification number: 10.5281/zenodo.8398688 Direct URL to data: https://zenodo.org/record/8398688

## 1 Value of the Data

• The dataset is useful to train human activity recognition systems, evaluate them using cross-subject validation approaches, and compare them.
• Mobile phone and smartwatch sensor data samples are simultaneously collected and annotated with human activity labels obtained via video recordings to establish ground truth.
• The twenty-three participants account for age diversity, ranging in ages from 23 to 66 years old, and gender balance, with 56%/44% male/female subjects, which allows further studies accounting for differences between age groups or gender.
• Researchers can use the dataset to compare smartphone- and smartwatch-based human activity recognition systems or to explore sensor fusion techniques on data from both devices.
• Recognition models can be trained with the collected dataset, including low-weight models to be deployed on a smartphone or smartwatch and executed in real time.

## 2 Data Description

**Fig. 1:**
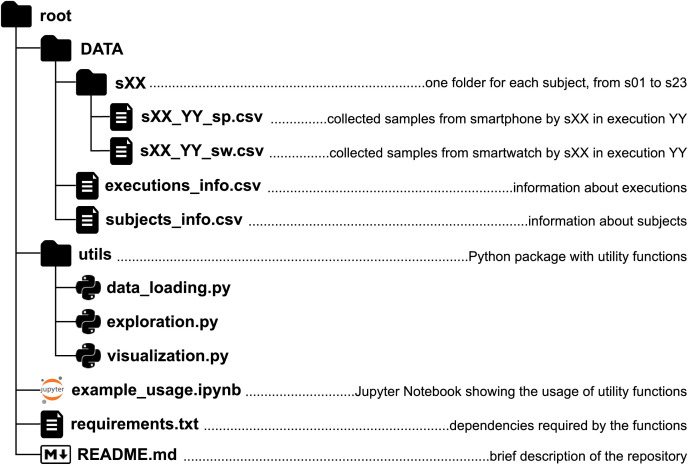
Repository structure

The dataset described in this article and associated files are stored in a repository whose structure is detailed in this section and depicted in Fig. 1. The repository is available on Zenodo [1].

The collected dataset is stored in the *DATA* directory of the repository and contains raw (i.e., no preprocessing steps applied) accelerometer and gyroscope samples from a smartphone and a smartwatch labelled with a certain human activity. Even though the labels are synchronized (see Section 3.2), the samples of each device are not synchronized with each other. In other words, whereas a label of an activity change at timestamp X refers to the same timestamp in both data streams, a data sample at timestamp Y in the smartphone data might not have an equivalent sample exactly at timestamp Y in the smartwatch data. Each subject executed the following specific sequence of human activities several times: being *seated* on a chair, *standing up* from a chair, *walking* three meters, *turning* around, *walking* back three meters, *turning* around, and *sitting down*). The *DATA* directory contains a subdirectory for each subject who participated in the data collection. Each subdirectory is named using the ID of the subject, which follows the format “sXX” (i.e., s01, s02, …, s23).

**Fig. 2:**
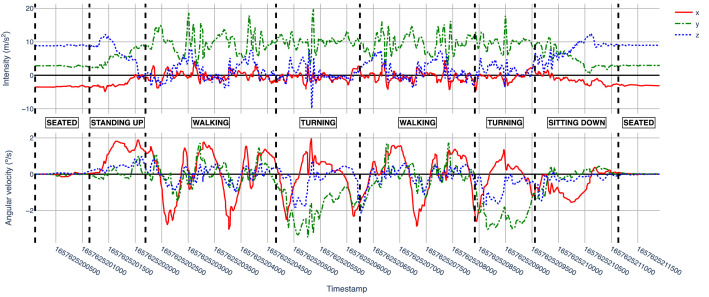
Smartphone collected accelerometer (top) and gyroscope (bottom) data by subject “s16” on his first execution (s16_01_sp.csv).

**Fig. 3:**
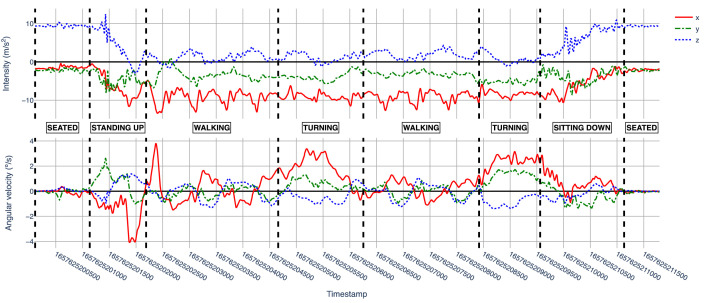
Smartwatch collected accelerometer (top) and gyroscope (bottom) data by subject “s16” on his first execution (s16_01_sw.csv).

Each subdirectory “sXX” contains *csv* files with the collected samples from the subject. The *csv* files are named using the pattern “sXX_YY_DEV'”, where “YY” is the number of the executed sequence, and “DEV” is the device used to collect the data (i.e., “sp” for the smartphone or “sw” for the smartwatch). Each row of a *csv* file contains a timestamped triaxial accelerometer and gyroscope sample, labelled with the corresponding human activity (i.e. ground truth). Table 1 describes each field in a row, which is the same structure and semantics for all subject data files. As an example of the type of data captured for one subject, Fig. 2, Fig. 3 show a plot of the accelerometer and gyroscope samples collected respectively from the smartphone and the smartwatch by the subject “s16” on his first execution (i.e., files “s16_01_sp.csv” and “s16_01_sw.csv”).

**Table 1:** Fields contained in each collected sample.

Column name	Column description
x_acc	Value of the accelerometer's *x* axis.
y_acc	Value of the accelerometer's *y* axis.
z_acc	Value of the accelerometer's *z* axis.
x_gyro	Value of the gyroscope's *x* axis.
y_gyro	Value of the gyroscope's *y* axis.
z_gyro	Value of the gyroscope's *z* axis.
timestamp	UNIX timestamp (milliseconds) when the sensor's data was collected.
label	Associated activity. One of SEATED, STANDING_UP, WALKING, TURNING or SITTING_DOWN

Even though the sampling rate used in the data collection applications was set to 100 Hz, Android applications are not always able to apply the requested sampling rate [2]. Therefore, based on the collected data, the average sampling rate was 102 Hz and 104 Hz for the smartphone and smartwatch, respectively. Table 2 summarizes the number of collected samples for each activity and device.

**Table 2:** Number of collected samples.

Device	SEATED	STANDING_UP	WALKING	TURNING	SITTING_DOWN	Total
Smartphone	32,764	27,303	115,069	52,209	31,868	259,213
Smartwatch	32,025	27,765	117,126	53,180	32,457	262,553

The *DATA* directory contains two additional files: subjects_info.csv, which contains information about the participants of the data collection; and executions_info.csv, which contains information about each sequence of activities performed. Table 3 and Table 4 describe the fields of each file.

**Table 3:** Fields contained in the subjects_info.csv file.

Column name	Column description
subject_id	ID of the subject (“sXX”).
age	Age of the subject.
gender	Gender of the subject.
height	Height (cm) of the subject.
weight	Weight (kg) of the subject.
dominant_hand	Dominant hand of the subject: R (right) or L (left).
executions	Number of activity sequences executed by the subject.

**Table 4:** Fields contained in the executions_info.csv file.

Column name	Column description
execution_id	ID of the sequence execution. Format: “sXX_YY”
orientation	Phone's orientation in the pocket. Four possible orientations: “front”, “front_inv”, “back” or “back_inv” (see Fig. 4).
first_turn	Direction of the first turn in the sequence: “l” for left, “r” for right.
second_turn	Direction of the second turn in the sequence: “l” for left, “r” for right.

In addition to the dataset, the repository includes a Python package named *utils*, which contains three utility modules with convenient functions for data exploration and visualization. Table 5 describes the contents of the package. The repository also contains a Jupyter Notebook file demonstrating how to use the package functions (*example-usage.ipynb*) and a file with the library dependencies required to execute those functions (*requirements.txt*). Finally, a *README.md* file briefly describes the structure and contents of the repository.

**Table 5:** Description of modules and functions contained in the utils package.

Module	Function	Description
data_loading	load_data()	Loads the dataset into a Python dictionary, where keys are activity sequence executions ids and values are the collected data.
	load_subjects_info()	Loads the subjects_info.csv into a Pandas DataFrame.
	load_executions_info()	Loads the executions_info.csv into a Pandas DataFrame.
exploration	count_samples()	Counts the number of collected samples per activity and device.
	subjects_age_range()	Provides statistics (mean, standard deviation, min, max) about subjects’ age.
	subjects_age_range_by_gender()	Provides statistics (mean, standard deviation, min, max) about subjects’ age grouped by gender.
	executions_by_gender()	Counts the number of activity sequences executed by gender.
visualization	plot_execution()	Plots the collected samples of a specified activity sequence execution. Used to generate Figs. 2 and 3.

## 3 Experimental Design, Materials and Methods

### 3.1 Subjects

Twenty-three physically healthy, white caucasian subjects (thirteen male, ten female) voluntarily participated in the data collection procedure. The mean age of the participants was 44.3 years with a standard deviation of 14.3 years, where the youngest and oldest subjects were 23 and 66 years old, respectively. The data collection procedure was explained to the subjects and informed written consent was obtained from them before starting the procedure. Table 6 shows the details of the subjects and the number of activity sequences executed.

**Table 6:** Details of the subjects and their number of activity sequences executed.

Subject ID	Age	Gender	Height (cm)	Weight (kg)	Dominant hand	Executions
s01	54	Male	190	83	Right	6
s02	31	Male	171	71	Right	9
s03	24	Female	161	62	Right	10
s04	51	Male	174	60	Right	10
s05	54	Male	172	85	Right	10
s06	53	Male	179	110	Right	10
s07	49	Male	176	88	Right	11
s08	63	Male	165	89	Right	9
s09	28	Female	164	49	Right	10
s10	66	Female	165	72	Right	10
s11	50	Male	181	70	Right	10
s12	46	Male	181	90	Right	10
s13	26	Male	170	65	Right	10
s14	34	Male	170	65	Right	10
s15	23	Female	166	60	Right	10
s16	25	Male	173	64	Left	10
s17	58	Female	156	53	Right	10
s18	61	Male	172	97	Right	10
s19	30	Female	160	58	Right	10
s20	58	Female	160	60	Right	10
s21	56	Female	160	55	Right	10
s22	31	Female	162	70	Right	9
s23	48	Female	174	78	Right	9

### 3.2 Devices

**Fig. 4:**
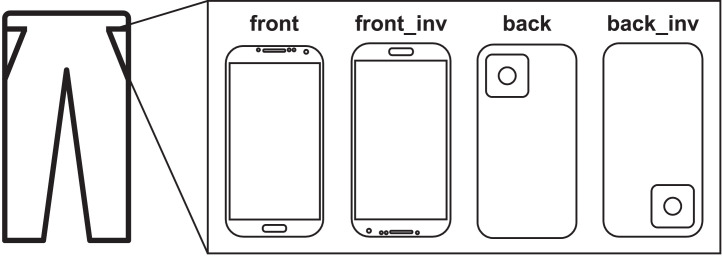
Different orientations of the smartphone placed in the pocket.

A Xiaomi Poco X3 Pro smartphone (M2102J20SG) and a TicWatch Pro 3 GPS smartwatch (WH12018), both equipped with a STMicroelectronics LSM6DSO IMU sensor, were used to collect accelerometer and gyroscope. The devices had a custom application installed (smartphone app [3], smartwatch app [4]) to collect the sensor samples at 100 Hz. The smartwatch was worn on the left wrist; the smartphone was carried in the front left trousers pocket, with an orientation chosen by the participant (see Fig. 4).

Another device, a Xiaomi Poco F2 Pro smartphone (M2004J11G), was used to video-record the subjects while performing the data collection procedure at 60 frames per second for data labelling (i.e., ground truth) purposes. Since three different devices were used for the data collection (i.e., smartphone, smartwatch and video-recording smartphone), small drifts on their internal clocks could exist, leading to inaccurate data labelling. Therefore, the Network Time Protocol (NTP) [5] was used to synchronize the internal clocks of the three devices.

### 3.3 Collection environment

The data collection was executed in a research laboratory at Universitat Jaume I. An obstacle-free, three-meter-long and two-meter-wide area with a flat ceramic floor and a combination of natural and artificial light was prepared to carry out the collection.

An armless chair was placed in on longitudinal extreme of the area and a visible floor mark was put in the opposite extreme. Thus, the chair and the floor mark were separated by three meters.

The environment was only occupied by a participant and a researcher to avoid any distraction or interference during the data collection. In addition to the smartphone used to video-record the collection and the personal devices of the participant, no other devices were enable in the environment that could interfere with the data collection process.

### 3.4 Experimental procedure

Each participant was asked to perform a specific sequence of activities (which corresponds with the TUG test [6]) starting from a seated position on a chair: standing up from the chair, walking three meters (indicated with a mark on the ground), turning around (180º), walking back to the chair, turning around (180º), and sitting down on the chair. The participants were free to choose the direction of their turns (i.e., left or right). Each participant was responsible for starting and stopping the data collection process for the sequence of activities, following the instructions below:

• Start data collection:
  ○ Press a “start” button on the smartphone application, lock the device and store it the left pocket.
  ○ Wait for a sound emitted from the smartphone.
  ○ Press a “start” button on the smartwatch application (already placed on the left wrist).
  ○ Wait for a vibration emitted from the smartwatch.
  ○ Start the sequence of activities.
• Stop data collection:
  ○ Finish the sequence of activities.
  ○ Press a “stop” button on the smartwatch application.
  ○ Get the phone from the pocket, unlock the device, and press a “stop” button on the smartphone application.

Each subject was instructed to perform the sequence of activities ten times, although some sequence executions were discarded due to non-compliance with the procedure (e.g., incorrect start of data collection, poor sequence execution, etc.). Table 6 shows the number of executions that each subject performed (“Executions” column), with a total of 223 executions.

Each activity sequence was video-recorded by a researcher. Then, each video was manually analyzed at frame level to determine the transitions between the executed activities and label the collected samples with the corresponding activity to establish the ground truth. The transitions and their identification criteria are the following:

• SEATED → STANDING_UP: determined when the participant's back separates from the chair's backrest.
• STANDING_UP → WALKING: determined when the participant's body is in an erect position and any of his/her feet starts to raise to execute a step.
• WALKING → TURNING: determined when the participant's shoulders or hip begin to rotate with respect to the center of the body.
• TURNING → WALKING: determined when the participant's shoulders or hip end the rotation, with the body facing to the chair and any feet starting to raise to execute a step.
• TURNING → SITTING_DOWN: determined when the participant begins to flex his/her knees and the higher body trunk bends forward.
• SITTING_DOWN → SEATED: determined when the participant's back contacts the chair's backrest.

For privacy reasons, the recorded videos are not publicly available.

## 4 Limitations

The main limitation of the data described in this article resides in the data labelling procedure. Data labelling was performed by visual inspection of videos recorded at 60 frames per second, which means that the time resolution of the video was 16.6 ms. However, sometimes, two adjacent frames were repeated, reducing the time resolution to 33.2 ms. On the other hand, the resolution of the sensors used for data collection was about 10 ms. Due to this resolution mismatch, there is a possible drift of up to three sensor samples, compared to the video recording. This could cause such samples, recorded during the transition from one activity to another, to be mislabelled. In addition, unintentional errors could have been introduced during the manual video-recording inspection and corresponding labelling process. Concerning the sampling rate, we note some minor variability which is imposed by the Android operating system and thus represents a real-world data collection process. Finally, while data heterogeneity w.r.t age and gender were ensured, there is an imbalance in handedness with most participants being right-handed.

## Ethics Statement

Informed written consent was obtained from all participants, and the data collection was approved by the ethics committee of the Universitat Jaume I (reference No. CD/88/2022) and carried out in accordance with the Declaration of Helsinki.

## CRediT Author Statement

**Miguel Matey-Sanz:** Conceptualization, Methodology, Software, Data Collection, Data Curation, Writing – Original Draft, Visualization. **Sven Casteleyn and Carlos Granell:** Conceptualization, Methodology, Resources, Writing – Review & Editing, Supervision, Funding acquisition.

## Data Availability

Smartphone and smartwatch inertial measurements from heterogeneous subjects for human activity recognition (Original data) (Zenodo)
